# Synthesis and In Vitro Characterization of Selective Cannabinoid CB2 Receptor Agonists: Biological Evaluation against Neuroblastoma Cancer Cells

**DOI:** 10.3390/molecules27093019

**Published:** 2022-05-07

**Authors:** Francesca Gado, Rebecca Ferrisi, Sarah Di Somma, Fabiana Napolitano, Kawthar A. Mohamed, Lesley A. Stevenson, Simona Rapposelli, Giuseppe Saccomanni, Giuseppe Portella, Roger G. Pertwee, Robert B. Laprairie, Anna Maria Malfitano, Clementina Manera

**Affiliations:** 1 Department of Pharmacy, University of Pisa, 56126 Pisa, Italy; francesca.gado@unimi.it (F.G.); rebecca.ferrisi@phd.unipi.it (R.F.); simona.rapposelli@unipi.it (S.R.); giuseppe.saccomanni@unipi.it (G.S.); 2 Department of Translational Medical Sciences, University of Naples Federico II, 80131 Napoli, Italy; sarah.disomma@unina.it (S.D.S.); fabiana.napolitano2@unina.it (F.N.); giuseppe.portella@unina.it (G.P.); 3 College of Pharmacy and Nutrition, University of Saskatchewan, Saskatoon, SK S7N 5E5, Canada; kam913@mail.usask.ca (K.A.M.); robert.laprairie@usask.ca (R.B.L.); 4 Institute of Medical Sciences, University of Aberdeen, Aberdeen AB25 2ZD, UK; l.a.stevenson@abdn.ac.uk (L.A.S.); rgp@abdn.ac.uk (R.G.P.); 5 Department of Pharmacology, College of Medicine, Dalhousie University, Halifax, NS B3H 4R2, Canada

**Keywords:** neuroblastoma, cannabinoid receptor 2, selective CB2R agonists

## Abstract

1,8-naphthyridine-3-carboxamide structures were previously identified as a promising scaffold from which to obtain CB2R agonists with anticancer and anti-inflammatory activity. This work describes the synthesis and functional characterization of new 1,8-naphthyridin-2(1*H*)-one-3-carboxamides with high affinity and selectivity for CB2R. The new compounds were able to pharmacologically modulate the cAMP response without modulating CB2R-dependent β-arrestin2 recruitment. These structures were also evaluated for their anti-cancer activity against SH-SY5Y and SK-N-BE cells. They were able to reduce the cell viability of both neuroblastoma cancer cell lines with micromolar potency (IC_50_ of **FG158a** = 11.8 μM and **FG160a** = 13.2 μM in SH-SY5Y cells) by a CB2R-mediated mechanism. Finally, in SH-SY5Y cells one of the newly synthesized compounds, **FG158a**, was able to modulate ERK1/2 expression by a CB2R-mediated effect, thus suggesting that this signaling pathway might be involved in its potential anti-cancer effect.

## 1. Introduction

Neuroblastoma (NB) is the most common solid extracranial tumor among children characterized by a severe mortality rate [1]. NB begins from immature nerve cells of the sympathetic nervous system and can lead to a wide range of clinical outcomes from spontaneous regression to incurable progression, with resistant therapy and poor prognosis [2,3]. Therefore, it is necessary to find novel therapeutic compounds that can achieve effective and efficient results against neuroblastomas.

Cannabinoid receptor 2 (CB2R), together with cannabinoid receptor 1 (CB1R), belongs to the endocannabinoid system (ECS), a relatively recently discovered physiological system known to play a fundamental role in establishing and maintaining human health [4]. CB1R is abundant in the central nervous system (CNS), where it plays a well-established role in regulating neuronal excitability [5]. In contrast, CB2R is predominantly expressed at the peripheral level and is involved in the anti-inflammatory effects of cannabinoids without causing psychotropic adverse effects, which are mainly associated with the stimulation of CB1R [6]. CB2R continues to be of interest because of its involvement in several pathological conditions including cancers. CB2R is reported to be upregulated in various tumor tissues, such as melanoma [7], bladder cancer [8], breast cancer [9], colon cancer [10], and hepatocellular carcinoma [11]. Many CB2R agonists have exhibited an antitumor effect in several cancers [12,13].

In a recent study reported in the literature, additional therapeutic targets against high-risk neuroblastoma were defined using an integrative data analysis combined with experimental evaluation in cell lines from patient-derived xenografts. In this work, CB2R was identified as one of the most promising targets for the treatment of high-risk neuroblastoma [14].

**Figure 1:**
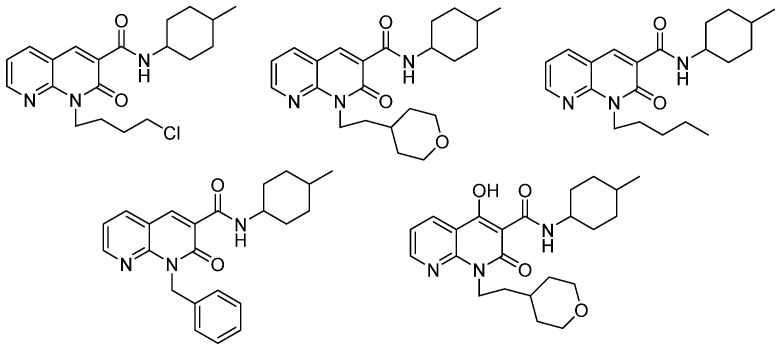
1,8-naphthyridin-2(1*H*)-one-3-carboxamide derivatives with antiproliferatve or/and anti-inflammatory activity.

Previously, we reported 1,8-naphthyridin-2(1*H*)-one-3-carboxamides as suitable scaffolds for the development of promising CB2R ligands with agonist behavior. Moreover, some of these compounds showed a CB2R-dependent anti-proliferative effect in various cancer cell lines and/or anti-inflammatory activity [15,16,17,18,19,20] (Figure 1).

Several 1,8-Naphthyridine derivatives have gained the attention of researchers for their anticancer properties and SAR studies on their efficacy as antitumor active compounds have been reported [21]. Furthermore, many other heterocyclic compounds such as indole, furano, isoxazole derivatives characterized by a carboxamide substituent have shown interesting anti-proliferative activity against different cancer cell lines [22,23,24,25].

**Figure 2:**
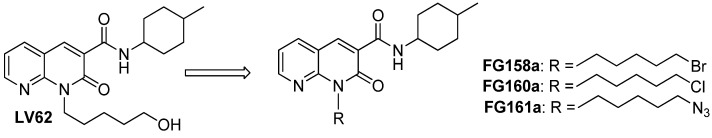
Design of the novel 1,8-naphthyridin-2(1*H*)-one-3-carboxamide derivatives.

The present work describes the synthesis of the new 1,8-naphthyridin-2(1*H*)-one-3-carboxamide derivatives **FG158a**, **FG160a**, and **FG161a** (Figure 2). The new compounds are structurally similar to the selective CB2R agonist N1-hydroxypentyl derivative **LV62** previously reported by us [26] but with the hydroxyl group being replaced by a bromine atom (**FG158a)**, a chlorine atom (**FG160a**), or an azido group (**FG161a)** (Figure 2). The effectiveness of the novel 1,8-naphthyridine-3-carboxamide derivatives and of compound **LV62** were determined against neuroblastoma cells.

## 2. Results and Discussion

### 2.1. Chemistry

#### Synthesis of Compounds **FG158a**, **FG160a**, and **FG161a**

**Scheme 1:**
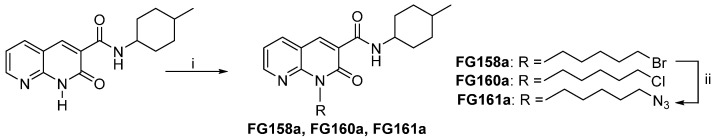
Reagents and conditions: (*i*) CsF, DMF, rt, 1 h; suitable R-Br or R-Cl, 30 °C, 24 h; (*ii*) NaN_3_, DMF, 60 °C, 12 h.

The synthetic route to obtain the new 1,8-naphthyridin-2(1H)-on-3-carboxamide derivatives **FG158a**, **FG160a**, and **FG161a** is outlined in Scheme 1. N-(4-methylcyclohexyl)-1,8-naphthyridin-2(1H)-on-3-carboxamide, prepared as previously reported in the literature [17], was subjected to a N-alkylation reaction by treatment with cesium fluoride in anhydrous DMF at room temperature for 1 h and then with 1,5-dibromopentane or 1-bromo-5-chloropentane at 30 °C for 24 h to yield the desired 1,8-naphthyridin-2-one derivatives **FG158a** and **FG160a**. The synthesis of the azide derivative **FG161a** included an additional step starting from compound **FG158a** with sodium azide at 60 °C for 12 h. Each crude mixture was purified by flash chromatography to produce **FG158a, FG160a**, and **FG161a**; in addition, the separation of the cis and trans isomers of each compound was achieved.

### 2.2. Biological Results

#### 2.2.1. [^3^H]CP55,940 Binding Assays

The binding affinities (K_i_ values) of the novel derivatives **FG158a**, **FG160a**, and **FG161a** and the compound **LV62** [26] as a mixture of *cis* and *trans* isomers (≅ 1:1) were evaluated at *h*CB1R and *h*CB2R using a [^3^H]CP55,940 radioligand displacement assay and membranes derived from Chinese hamster ovary (CHO) cells stably expressing either receptor. The results are summarized in Table 1. The non-selective orthosteric CBR ligand CP55,490 was used as a reference compound. At *h*CB2R, the new compounds **FG158a**, **FG160a**, and **FG161a** fully displaced [^3^H]CP55,940, proving that they are high-affinity CB2R ligands (K_i_ values from 45 nM to 16.5 nM) analogously to the reference compound **LV62** (K_i_ = 37 nM) (Table 1). At *h*CB1R, the new compounds **FG158a**, **FG160a**, **FG161a**, and **LV62** were not able to displace [^3^H]CP55,940, indicating no affinity of these ligands for *h*CB1R, and hence high CB2R selectivity (Table 1). These results showed that the replacement of the hydroxy group of the hydroxypentyl substituent in position 1 of **LV62** by the bromine, chloro, or azido group made it possible to maintain high affinity and selectivity for CB2R. This finding is in accordance with the result obtained previously by replacement of the hydroxy group of **LV62** by a fluorine atom [26].

#### 2.2.2. CB2R Functional Activity: cAMP and β-Arrestin2 Assays

**Figure 3:**
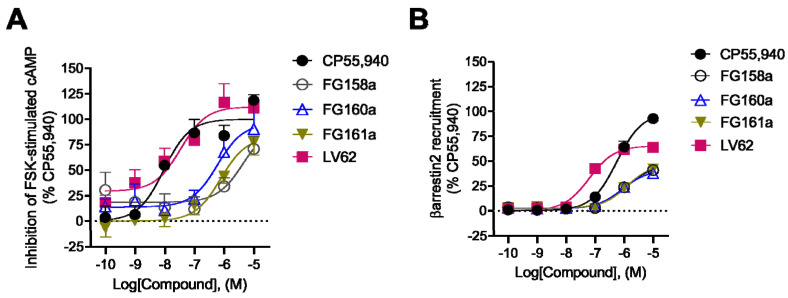
CB2R-dependent inhibition of FSK-stimulated cAMP and CB2R−dependent recruitment of βarrestin2. CHO cells stably expressing *h*CB2R were treated with 0.10 nM−10 μM compounds for 90 min and cAMP inhibition (**A**) or βarrestin2 recruitment (**B**) was measured. cAMP and βarrestin2 recruitment data are expressed as % CP55,940 response. Data are fitted to a non−linear regression (three parameter model, GraphPad v. 9.0). Data are mean ± S.E.M. of 6 independent experiments performed in triplicate.

Following the initial [^3^H]CP55,940 binding assessment, **FG158a**, **FG160a**, **FG161a**, and **LV62** (as a mixture of cis and trans isomers) were evaluated for their functional activity to modulate the Gα_i/o_-dependent inhibition of forskolin (FSK)-stimulated cAMP accumulation and β-arrestin2 recruitment in CHO cells stably expressing *h*CB2R. The non-selective orthosteric CBR ligand CP55,490 was used as a reference compound. For the cAMP inhibition assay, cells were treated with 10 μM FSK and synthetized compounds for 90 min to assess compound concentration-dependent activity. The results showed that **LV62** displayed the highest potency (34 (3.9–220)) nM and efficacy (110 ± 8.8). (Table 1). **FG160a** and **FG161a** were able to modulate the cAMP response but their potency and efficacy were lower than those of the compound **LV62** (Table 1). Conversely, compound **FG158** proved not to affect this signaling pathway (Figure 3A; Table 1).

Compounds **FG158a**, **FG160a**, **FG161a**, and **LV62** were also evaluated for their ability to recruit β-arrestin2, since G protein-coupled receptors also interact with β-arrestin, which facilitates receptor internalization, recycling, degradation, and signaling. Interestingly, the trend relating the potency and efficacy values for compounds **FG158a**, **FG160a**, and **FG161a** suggests that these were not able to modulate β-arrestin2 recruitment (EC_50_ > 10,000 nM) (Figure 3A; Table 1). In contrast, compound **LV62** produced an increase in the β-arrestin2 recruitment (Figure 3B; Table 1).

#### 2.2.3. Investigation of Toxicity Levels against the Neuroblastoma Cells

**Figure 4:**
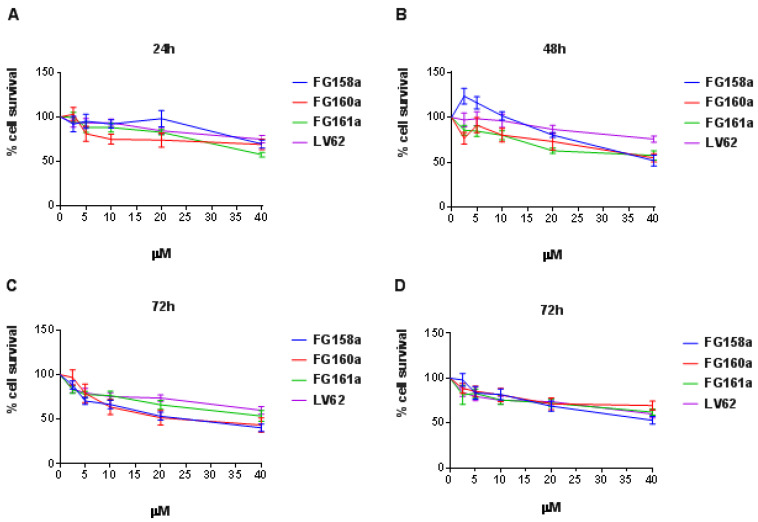
Inhibition of cell viability. The effect on cell viability by CB2R agonists was determined by sulforhodamine B (SRB) assays in SH-SY5Y and SK-N-BE cell lines. SH-SY5Y cells were treated with the compounds indicated in the figure for 24 h (**A**), 48 h (**B**), and 72 h (**C**) at the concentrations reported in the histograms. SK-N-BE cells were treated with the same compounds for 72 h (**D**). Figures report cell viability (mean of at least 3 independent experiments ± SEM) as percent of cell survival vs. untreated cells (considered as 100% of survival). In SH-SY5Y cells after 72 h of treatment, the IC_50_ values were: **FG158a** = 11.8 µM; **FG160a** = 13.2 µM, **FG161a** = 27.53 µM, **LV62** = 38.11 µM. The statistical significance was calculated with two-way ANOVA using Dunnett’s multiple comparisons test.

The cytotoxic anticancer activity of CBR agonists has been suggested by several studies [12,13]. The selective CB2R agonist JWH-133 inhibits SH-SY5Y cell proliferation, presumably via a CB2R-independent mechanism [27]. We tested the anti-proliferative activity of the novel CB2R agonists **FG158a**, **FG160a, FG161a**, and **LV62** in neuroblastoma cell lines (Figure 4). The compounds were assayed as a mixture of *cis* and *trans* isomers. We observed that in SH-SY5Y cells after 24 h (Figure 4A), 48 h (Figure 4B), and 72 h (Figure 4C) of treatment, **FG158a**, **FG160a**, **FG161a**, and **LV62** inhibited cell viability. After 72 h of treatment, the effect was higher and could also be observed at lower concentrations. The inhibitory effect was confirmed in another neuroblastoma cell line, SK-N-BE cells. After 72 h of treatment, the compounds inhibited cell viability (Figure 4D).

**Figure 5:**
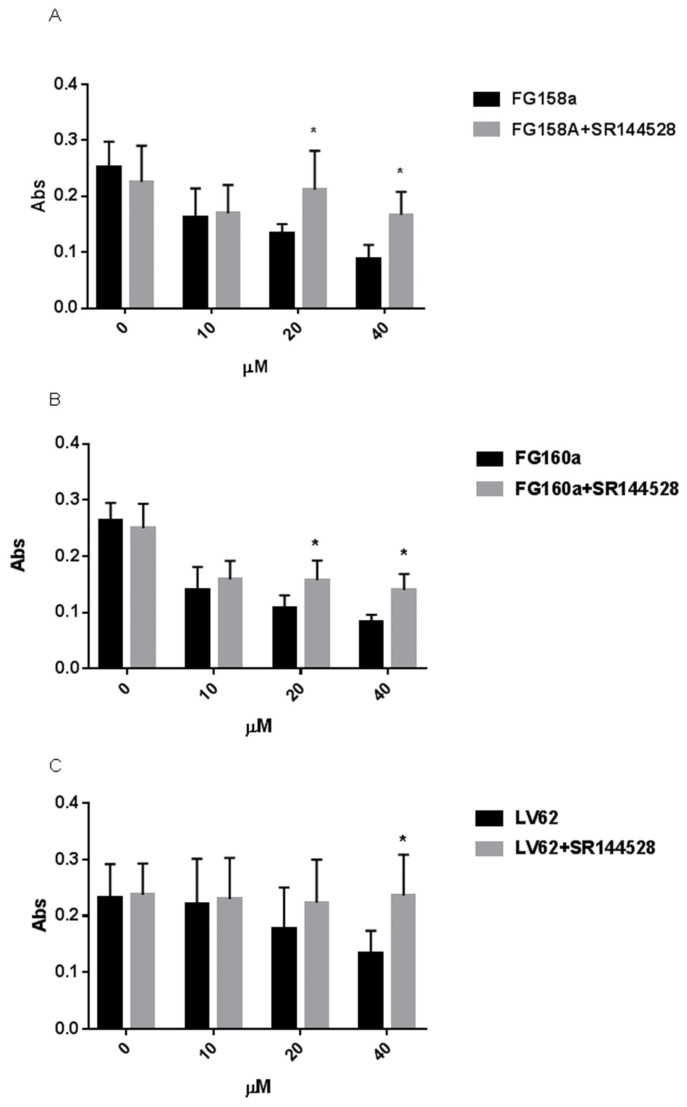
CB2R–mediated effect on cell viability. The effect on cell viability by selected CB2R agonists (**FG158a**, **FG160a**, and **LV62**) at the concentrations reported in the histograms was explored in the presence of SR144528 (1 µM added 1 h before the CB2R agonists) after 72 h of treatment. Figures (**A**–**C**) report cell viability (mean of 3 independent experiments) expressed as absorbance (Abs). The statistical significance was calculated with two-way ANOVA using Tukey’s multiple comparisons test (* *p* ≤ 0.05).

To address a CB2R-mediated mechanism, we tested **FG158a**, **LV62**, and **FG160a** at inhibitory concentrations in SH-SY5Y cells pre-incubated with the CB2R antagonist SR144528. After 72 h of treatment, we observed a reversion in cell viability at 20 and 40 µM of **FG158a** and **FG160a** in the presence of SR144528. The inhibitory effect of **LV62** was reverted only at 40 µM (Figure 5). These data suggest that CB2R agonists can arrest neuroblastoma cell viability and that their effect is mediated by CB2R.

#### 2.2.4. Effect of **FG158a** on ERK1/2

**Figure 6:**
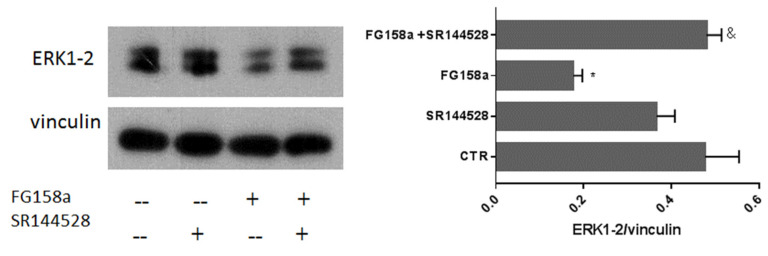
**FG158a** inhibition of ERK1/2 is mediated by CB2R. Western blot and densitometric analysis of ERK protein is shown for **FG158a** treated SH−SY5Y cells and the combined effect with SR144528 is also shown. Vinculin was used as a control for normalization. The blots are representative of at least 3 independent experiments and the densitometric analysis reports the mean of the experiments. The statistical analysis was performed by parametric two tailed t-test (* *p* < 0.05 **FG158a** calculated with respect to the CTR; ^&^ *p* < 0.01 **FG158a**+SR144528 calculated with respect to **FG158a**).

Among the CB2R agonists tested, we selected **FG158a** as it provides the best dose–response curve in SH-SY5Y cells (Figure 4B). The effect of CB2R agonists on the ERK pathway has been previously detected in other tumor models such as a breast cancer model [28]. The dysregulation of the ERK/MAPK pathway is crucial in neuroblastoma. In neuroblastoma primary cells, the ERK/MAPK pathway has been observed to be highly activated [29]. We addressed the effect of **FG158a** on MAPK kinase activation by the evaluation of its component ERK1/2 in the presence and in the absence of the antagonist SR144528 (Figure 6). We observed that 20 µM **FG158a** inhibited ERK1/2 expression with respect to the untreated control cells, whereas the antagonist SR144528 reversed the inhibitory effect of the agonist. These data demonstrate the activity of **FG158a** on ERK1/2 levels and that the effect is mediated by CB2R.

## 3. Material and Methods

### 3.1. Chemistry

Commercially available reagents were purchased from Merk Life Science (Milano, Italy) or Fluorochem (Glossop, UK) and used without purification. ^1^H NMR and ^13^C NMR were recorded at 400 and 100 MHz, respectively, on a Bruker AVANCE IIITM 400 spectrometer (Mannheim, Germany). Chemical shift (δ) is reported in parts per million related to the residual solvent signal, while coupling constants (J) are expressed in Hertz (Hz). Organic solutions were dried over anhydrous Na_2_SO_4_. Evaporation was carried out in vacuo using a rotating evaporator. Silica gel flash chromatography was performed using silica gel 60 Å (0.040–0.063 mm; Merck Life Science S.r.l., Milano, Italy). Reactions were monitored by TLC on Kieselgel 60 F254 (Merck Life Science S.r.l., Milano, Italy) with detection by UV light (λ = 254 nm). All reactions involving air- or moisture-sensitive reagents were performed under nitrogen atmosphere using anhydrous solvents. Melting points were determined on a Kofler hot-stage apparatus and are uncorrected. Elemental analyses were performed in our analytical laboratory and agreed with theoretical values to within ± 0.4%.

#### 3.1.1. Synthesis of 1-(5-bromopentyl)-N-(4-methylcyclohexyl)-2-oxo-1,2-dihydro-1,8-naphthyridine-3-carboxamide (**FG158a**)

Cesium fluoride (798 mg, 5.25 mmol) was added to a solution of N-(4-methylcyclohexyl)-1,8-naphthyridin-2(1*H*)-on-3-carboxamide [17] (500 mg, 1.75 mmol) in anhydrous DMF (5.25 mL). The solution was stirred at room temperature for about one hour and then treated with 1,5-dibromopentane or 1-bromo-5-chloropentane (0.72 mL, 5.25 mmol). The reaction mixture was stirred at 30 °C for 24 h. After that, the solvent was removed under reduced pressure and the obtained residue was dissolved in CHCl_3_ and washed with water. The organic phase was dried over Na_2_SO_4_, filtered, and evaporated under reduced pressure yielding a crude product that was purified by flash column chromatography using hexane/AcOEt 5:5 to yield the desired 1,8-naphthyridine-3-carboxamides **FG158a** as a *cis*/*trans* diastereoisomeric mixture. The separation of *cis* and *trans* isomers was also achieved. **FG158a:** Yield: 46%. ^1^H-NMR (CDCl_3_) δ 10.01 and 9.63 (2d, 1H, *J* = 7.2 Hz, NH); 8.86 (s, 1H, H_4_); 8.71 (dd, 1H, *J* = 4.6 Hz, 2.0 Hz, H_7_); 8.08 (dd, 1H, *J* = 7.6 Hz, 2.0 Hz, H_5_); 7.28 (dd, 1H, *J* = 7.6 Hz, 4.6 Hz, H_6_); 4.59 (t, 2H, *J* = 7.8 Hz, NCH_2_); 4.27 and 3.92 (2m, 1H, CH); 3.44 (t, 2H, *J* = 6.8 Hz, CH_2_Br); 2.10–1.09 (m, 15H, cyclohexyl + 3xCH_2_); 0.98 and 0.91 (2d, 3H, *J* = 6.8 Hz, CH_3_). ^13^C-NMR (CDCl_3_) *δ* 162.84, 162.06, 152.13, 149.78, 142.07, 138.64, 123.36, 119.21, 115.19, 49.03, 45.91, 41.35, 34.12, 33.18, 32.20, 31.47, 30.63, 30.48, 29.66, 27.43, 25.74, 22.81, 21.66. Anal. Calcd. for C_21_H_28_BrN_3_O_2_; C, 58.07; H, 6.50; N, 9.67; Found C, 58.23; H, 6.42; N, 9.72. **FG158a**-***trans***: Yield 16%; mp 121–123 °C. ^1^H-NMR (CDCl_3_) δ 9.63 (d, 1H, *J* = 7.2 Hz, NH); 8.86 (s, 1H, H_4_); 8.71 (dd, 1H, *J* = 4.6 Hz, 2.0 Hz, H_7_); 8.08 (dd, 1H, *J* = 7.6 Hz, 2.0 Hz, H_5_); 7.28 (dd, 1H, *J* = 7.6 Hz, 4.6 Hz, H_6_); 4.59 (t, 2H, *J* = 7.8 Hz, NCH_2_); 3.92 (m, 1H, CH); 3.44 (t, 2H, *J* = 6.6 Hz, CH_2_Br); 2.10–1.09 (m, 15H, cyclohexyl + 3xCH_2_); 0.91 (d, 3H, *J* = 6.8 Hz, CH_3_). ^13^C-NMR (CDCl_3_) *δ* 163.16, 162.55, 152.66, 150.30, 142.48, 139.07, 123.80, 119.66, 115.59, 49.43, 42.24, 34.55, 33.62, 33.01, 32.64, 31.58, 27.61, 26.28, 22.89. Anal. Calcd. for C_21_H_28_BrN_3_O_2_; C, 58.07; H, 6.50; N, 9.67; Found C, 58.15; H, 6.57; N, 9.70. **FG158a**-***cis***: Yield 21%; mp 116–118 °C. ^1^H-NMR (CDCl_3_) δ 10.01 (d, 1H, *J* = 7.2 Hz, NH); 8.86 (s, 1H, H_4_); 8.71 (dd, 1H, *J* = 4.6 Hz; 2.0 Hz, H_7_); 8.08 (dd, 1H, *J* = 7.6 Hz; 2.0 Hz, H_5_); 7.28 (dd, 1H, *J* = 7.6 Hz; 4.6 Hz, H_6_); 4.59 (t, 2H, *J* = 7.8 Hz, NCH_2_); 4.27 (m, 1H, CH); 3.44 (t, 2H, *J* = 6.6 Hz, CH_2_Br); 2.03–1.28 (m, 15H, cyclohexyl + 3xCH_2_); 0.98 (d, 3H, *J* = 6.8 Hz, CH_3_). ^13^C-NMR (CDCl_3_) *δ* 162.53, 161.88, 151.97, 149.68, 141.66, 138.44, 123.19, 118.96, 114.89, 45.62, 41.47, 33.65, 32.35, 31.03, 30.18, 29.58, 26.93, 25.59, 21.51. Anal. Calcd. for C_21_H_28_BrN_3_O_2_; C, 58.07; H, 6.50; N, 9.67; Found C, 58.33; H, 6.37; N, 9.55.

#### 3.1.2. Synthesis of 1-(5-chloropentyl)-N-(4-methylcyclohexyl)-2-oxo-1,2-dihydro-1,8-naphthyridine-3-carboxamide (**FG160a**)

Compound **FG160a** was prepared as described for compound **FG158** using 1-bromo-5-chloropentane and purified by flash column chromatography on silica gel using hexane/AcOEt 5:5 to yield the desired 1,8-naphthyridine-3-carboxamides **FG160a** as a *cis*/*trans* diastereoisomeric mixture. The separation of *cis* and *trans* isomers was also achieved. **FG160a**: Yield: 44%. ^1^H-NMR (CDCl_3_) δ 10.01 and 9.63 (2d, 1H, *J* = 7.2 Hz, NH); 8.86 (s, 1H, H_4_); 8.71 (dd, 1H, *J* = 4.8 Hz, 1.6 Hz, H_7_); 8.07 (dd, 1H, *J* = 7.6 Hz, 1.6 Hz, H_5_); 7.28 (dd, 1H, *J* = 7.6 Hz, 4.8 Hz, H_6_); 4.60 (2t, 2H, *J* = 7.8 Hz, NCH_2_); 4.25 and 3.91 (2m, 1H, CH); 3.58 (2t, 2H, *J* = 6.8 Hz, CH_2_Cl); 2.10–1.09 (m, 15H, cyclohexyl + 3xCH_2_); 0.97 and 0.91 (2d, 3H, *J* = 6.6 Hz, CH_3_). ^13^C-NMR (CDCl_3_) *δ* 162.53, 161.93, 152.03, 149.66, 141.76, 138.38, 123.20, 119.00, 114.93, 48.79, 44.93,45,70, 44.93, 41.65, 41.55, 33.91, 32.98, 32.24, 32.01, 30.20, 29.59, 27.11, 24.37, 24.34, 22.26, 21.49. Anal. Calcd. for C_21_H_28_ClN_3_O_2_; C, 64.69; H, 7.24; N, 10.78; Found C, 64.44; H, 7.15; N, 10.65. **FG-160a**-***trans***: Yield 18%; mp 124–126 °C. ^1^H-NMR (CDCl_3_) δ 9.63 (d, 1H, *J* = 7.2 Hz, NH); 8.86 (s, 1H, H_4_); 8.71 (dd, 1H, *J* = 4.8 Hz, 1.6 Hz, H_7_); 8.07 (dd, 1H, *J* = 7.6 Hz, 1.6 Hz, H_5_); 7.28 (dd, 1H, *J* = 7.6 Hz, 4.8 Hz, H_6_); 4.58 (t, 2H, *J* = 7.8 Hz, NCH_2_); 3.91 (m, 1H, CH); 3.57 (t, 2H, *J* = 6.8 Hz, CH_2_Cl); 2.10–1.09 (m, 15H, cyclohexyl + 3xCH_2_); 0.91 (d, 3H, *J* = 6.6 Hz, CH_3_). ^13^C-NMR (CDCl_3_) *δ* 162.58, 161.98, 152.09, 149.72, 141.90, 138.49, 123.21, 119.08, 115.00, 48.85, 44.94, 41.69, 33.98, 33.04, 32.29, 32.06, 27.17, 24.43, 22.31. Anal. Calcd. for C_21_H_28_ClN_3_O_2_; C, 64.69; H, 7.24; N, 10.78; Found C, 64.71; H, 7.35; N, 10.85. **FG-160a**-***cis***: Yield 22%; mp 104–106 °C. ^1^H-NMR (CDCl_3_) δ 10.01 (d, 1H, *J* = 7.2 Hz, NH); 8.86 (s, 1H, H_4_); 8.70 (dd, 1H, *J* = 4.6 Hz; 1.6 Hz, H_7_); 8.07 (dd, 1H, *J* = 7.6 Hz; 1.6 Hz, H_5_); 7.28 (dd, 1H, *J* = 7.6 Hz; 4.6 Hz, H_6_); 4.60 (t, 2H, *J* = 7.8 Hz, NCH_2_); 4.25 (m, 1H, CH); 3.58 (t, 2H, *J* = 6.8 Hz, CH_2_Cl); 1.93–1.27 (m, 15H, cyclohexyl + 3xCH_2_); 0.97 (d, 3H, *J* = 6.6 Hz, CH_3_). ^13^C-NMR (CDCl_3_) *δ* 162.61, 161.97, 152.03, 149.76, 141.71, 138.44, 123.27, 119.01, 114.96, 45.70, 44.95, 41.57, 32.27, 31.09, 30.25, 29.64, 27.14, 24.37, 21.55. Anal. Calcd. for C_21_H_28_ClN_3_O_2_; C, 64.69; H, 7.24; N, 10.78; Found C, 64.60; H, 7.19; N, 10.84.

#### 3.1.3. Synthesis of 1-(5-azidopentyl)-N-(4-methylcyclohexyl)-2-oxo-1,2-dihydro-1,8-naphthyridine-3-carboxamide (**FG161a**)

A mixture of the bromo derivative **FG158a** (0.20 g 0.46 mmol) and NaN_3_ (89.7 mg, 1.38 mmol) in anhydrous DMF (5.0 mL) was heated in a sealed vial at 60 °C for 12 h. After cooling, the solvent was removed under reduced pressure and the residue was treated with water and repeatedly extracted with dichloromethane. The organic phases were collected, dried over Na_2_SO_4_, filtered, and evaporated under reduced pressure to produce a solid residue that was purified by flash column chromatography using using hexane/AcOEt 4:6 to yield **FG161a** as a *cis*/*trans* diastereoisomeric mixture. The separation of *cis* and *trans* isomers was also achieved. ^1^H- and ^13^C-NMR Spectra of compounds **FG158a**-*trans*, **FG158a**-*cis* are shown in Figures S2 and S3. **FG161a:** Yield 52%. ^1^H-NMR (CDCl_3_) δ 10.00 and 9.64 (2d, 1H, *J* = 7.6 Hz, NH); 8.86 (s, 1H, H_4_); 8.70 (dd, 1H, *J* = 4.6 Hz, 1.8 Hz, H_7_); 8.08 (dd, 1H, *J* = 7.8 Hz, 1.8 Hz, H_5_); 7.28 (dd, 1H, *J* = 7.8 Hz, 4.6 Hz, H_6_); 4.60 (2t, 2H, *J* = 7.6 Hz, NCH_2_); 4.25 and 3.92 (2m, 1H, CH); 3.31 (2t, 2H, *J* = 6.8 Hz, CH_2_N_3_); 2.10–1.08 (m, 15H, cyclohexyl + 3xCH_2_); 0.98 and 0.92 (2d, 3H, *J* = 6.4 Hz, CH_3_). ^13^C-NMR (CDCl_3_) *δ* 162.45, 161.82, 151.91, 149.60, 141.66, 138.32, 123.10, 118.90, 114.84, 51.43, 48.52, 45.73, 41.49, 33.64, 32.70, 31.73, 31.10, 30.27, 29.66, 28.48, 27.26, 24.09, 21.97, 21.56. Anal. Calcd. for C_21_H_28_N_6_O_2_; C, 63.62; H, 7.12; N, 21.20; Found C, 63.51; H, 7.02; N, 21.07. **FG161**-*trans*: Yield 16%; mp 115–117 °C. ^1^H-NMR (CDCl_3_) δ 9.64 (bd, 1H, NH, *J* = 8.0 Hz); 8.86 (s, 1H, H_4_); 8.70 (dd, 1H, *J* = 4.6 Hz, 1.8 Hz, H_7_); 8.08 (dd, 1H, *J* = 7.8 Hz, 1.8 Hz, H_5_); 7.28 (dd, 1H, *J* = 7.8 Hz, 4.6 Hz, H_6_); 4.58 (t, 2H, *J* = 7.6 Hz, NCH_2_); 3.92 (m, 1H, CH); 3.30 (t, 2H, *J* = 6.8 Hz, CH_2_N_3_); 2.10–1.08 (m, 15H, cyclohexyl + 3xCH_2_); 0.92 (d, 3H, *J* = 6.4 Hz, CH_3_). ^13^C-NMR (CDCl_3_) *δ* 162.26, 161.65, 151.76, 149.40, 141.58, 138.16, 122.88, 118.75, 114.68, 51.06, 48.52, 41.36, 33.64, 32.70, 31.73, 28.32, 27.10, 23.94, 21.97. Anal. Calcd. for C_21_H_28_N_6_O_2_; C, 63.62; H, 7.12; N, 21.20; Found C, 63.55; H, 7.22; N, 21.37. **FG161-*cis***: Yeld 19%; mp 111–113 °C. ^1^H-NMR (CDCl_3_) δ 10.00 (bd, 1H, NH, *J* = 7.6 Hz); 8.87 (s, 1H, H_4_); 8.70 (dd, 1H, *J* = 4.6 Hz; 1.8 Hz, H_7_); 8.07 (dd, 1H, *J* = 7.8 Hz; 1.8 Hz, H_5_); 7.27 (dd, 1H, *J* = 7.8 Hz; 4.6 Hz, H_6_); 4.60 (t, 2H, *J* = 7.6 Hz, NCH_2_); 4.25 (m, 1H, CH); 3.31 (t, 2H, *J* = 6.8 Hz, CH_2_N_3_); 1.86–1.25 (m, 15H, cyclohexyl + 3xCH_2_); 0.98 (d, 3H, *J* = 6.4 Hz, CH_3_). ^13^C-NMR (CDCl_3_) *δ* 162.64, 161.99, 152.06, 149.80, 141.74, 138.47, 123.31, 119.04, 114.99, 51.43, 45.73, 41.61, 31.10, 30.27, 29.66, 28.66, 27.42, 24.24, 21.56. Anal. Calcd. for C_21_H_28_N_6_O_2_; C, 63.62; H, 7.12; N, 21.20; Found C, 63.82; H, 7.19; N, 21.36.

### 3.2. Biological Assays

#### 3.2.1. Reagents and Cell Lines

CP55,940 was purchased from Cayman Chemicals (Ann Arbor, MI, USA). [^3^H]CP55,940 (174.6 Ci/mmol) was obtained from PerkinElmer (Guelph, ON, Canada). CHO cells stably expressing *h*CB1R or *h*CB2R were maintained at 37 °C and 5% CO_2_ in Gibco Ham’s F-12 nutrient mix (Fisher Scientific, Loughborough, UK ) supplemented with 2 mM L-glutamine, 10% fetal bovine serum (FBS), 0.6% penicillin−streptomycin (Fisher Scientific, Loughborough UK), and the disulfate salt of geneticin (G418) as previously described (Sigma-Aldrich, Poole, UK) [24]. For subsequent radioligand displacement assays, membranes were prepared by scraping cells from flasks, centrifuging the cells, and storing the cell pellet at −20 °C. When required for us, cell pellets were defrosted and diluted in tris buffer (50 mM Tris-HCl and 50 mM Tris-base). As we have described in previous studies, HitHunter (cAMP) and PathHunter (βarrestin2) CHO-K1 cells stably expressing *h*CB1R or *h*CB2R from DiscoveRx^®^ (Eurofins, Fremont, CA, USA) were maintained at 37 °C, 5% CO_2_ in F-12 DMEM containing 10% FBS and 1% Pen/Strep with 800 µg/mL geneticin (HitHunter) or 800 µg/mL G418 and 300 µg/mL hygromycin B (PathHunter) [30].

Human neuroblastoma SHSY5Y and SK-N-BE cell lines were grown in DMEM (GIBCO, Paisley, UK) and MEM (Sigma-Aldrich, St.Louis, MO, USA), respectively, supplemented with 2 mM l-glutamine, 50 ng/mL streptomycin, 50 units/mL penicillin and 10% heat-inactivated fetal bovine serum (FBS) in a humidified atmosphere (5% CO_2_ at 37 °C). Cells were detached as previously described [31] with 0.25% trypsin (Sigma-Aldrich) at 70–80% confluence.

#### 3.2.2. Radioligand Displacement Assay

We have previously described radioligand displacement assays in detail [30]. Briefly, 0.7 nM [^3^H]CP55,940, tris binding buffer (50 mM Tris-HCl, 50 mM Tri-base, 0.1% BSA, pH 7.4), and 50 μg *h*CB1R or *h*CB2R cell membranes were used to a total assay volume of 500 μL per reaction at 37 °C for 60 min. Binding reactions were terminated by ice-cold tris binding buffer and subsequent vacuum filtration (24-well sampling manifold (Brandel cell harvester; Brandel Inc., Gaithersburg, MD, USA)) using Brandel GF/B filters soaked in wash buffer at 4 °C for at least 24 h. Filters were washed with 1.2 mL of tris-binding buffer six times, then oven-dried for 60 min before being incubated in 3 mL of scintillation fluid (Ultima Gold XR, PerkinElmer, Seer Green, Buckinghamshire, UK). Radioactivity was quantified by liquid scintillation spectrometry. All compounds were first prepared at 10 mM solutions in DMSO. The final vehicle concentration for assays was 0.1% DMSO. All assays were performed in duplicate.

#### 3.2.3. HitHunter^®^ cAMP Assay

The quantification of FSK-stimulated cAMP accumulation using the DiscoveRx HitHunter assay (DiscoveRx, Eurofins, Fremont, CA, USA) has been described in detail previously elsewhere [30]. Briefly, 20,000 cells/well were plated in low-volume 96-well plates and incubated overnight in Opti-MEM with 1% FBS at 37 °C and 5% CO_2_. The next day, the media were replaced with cell assay buffer (DiscoveRx). Immediately after this, the cells were simultaneously treated with 10 µM FSK and ligands for 90 min. Subsequent additions of cAMP antibody solution and cAMP working detection solutions followed the manufacturer’s directions (DiscoveRx) and cells were stored for 60 min at room temperature. cAMP solution A (DiscoveRx) was added according to the manufacturer’s directions followed by another 60 min room temperature incubation prior to chemiluminescence being measured on a Cytation5 plate reader (top read, gain 200, integration time 10,000 ms).

#### 3.2.4. PathHunter^®^ CB1R β-Arrestin2 Assay

The quantification of β-arrestin2 recruitment using the DiscoveRx PathHunter assay has been described by our group previously and is summarized here. [30]. Twenty thousand cells/well were grown overnight in Opti-MEM containing 1% FBS at 37 °C and 5% CO_2_ in low-volume 96 well plates. The next day, cells were treated with compounds for 90 min at 37 °C. Detection solution was added to cells according to the manufacturer’s directions (DiscoveRx) and cells were stored for 60 min at room temperature. After this incubation, chemiluminescence was quantified on a Cytation5 plate reader (top read, gain 200, integration time 10,000 ms).

#### 3.2.5. Sulforhodamine B (SRB) Assay

Cells seeded for 24 h in triplicate in 96-well plates at a density of 8000 cells/well were left to adhere overnight. CB2R agonists were added to the culture in triplicate at concentrations ranging from 2.5–40 µM. After 24 h, 48 h, and 72 h, cells were fixed, shaking for 2 h at 4 °C with 50% *v*/*v* trichloroacetic acid and washed with distilled water. Cells left overnight to dry were stained with 0.4% *w*/*v* SRB in 1% *v*/*v* acetic acid (at room temperature for 30 min on a shaker) and then washed with 1% acetic acid to allow for the removal of the unbound dye. TRIS-HCl 10 mM, pH 7.4 was added to the cells and absorbance was measured at 495 nm as previously shown [32,33] using a Glomax^®^ Discover Microplate Reader (Promega, Madison, WI, USA). In combinatory assays, similar procedures were adopted and the CB2R antagonist SR144528 (1 µM) was added 1 h before the CB2 agonists (10, 20 and 40 µM) to SHSY5Y cells that were harvested as described above after 72 h of incubation.

#### 3.2.6. Electrophoresis and Immunoblots

After the treatment with **FG158a** (20 μM) in the presence and absence of SR144528 (added 1 h before the CB2R agonist at the concentration of 1 µM), SHSY5Y cells were centrifuged and cell pellets were lysed in R.I.P.A. buffer (50 mM Tris-HCl, pH = 7.4; 150 mM NaCl; 0.5M EDTA, 1% NP-40; 0.5% sodium deoxycholate; 0.1% SDS; 1:100 phosphatase and protease inhibitors, both added before the lysis). Samples were centrifuged (17,900× *g* 20′ at 4 °C) and protein concentration was determined by Bio-Rad Protein Assay (Bio-Rad, Berkeley, CA, USA) as previously described [34]. Lysates (30 μg of proteins) re-suspended in Laemmli sample buffer were electrophoresed on 10% SDS-polyacrylamide gel. Samples were resolved under constant voltage (100 mA) and transferred to PVDF membranes (Millipore Corporation, Darmstadt, Germany). Blots were blocked with 5% BSA in TBS containing 0.1% Tween-20 for 1 h at room temperature. Filters were incubated overnight at 4 °C with 1:1000 dilution of ERK1/2 (SC-514302, Santa Cruz Biotechnology Inc., Santa Cruz, CA, USA) and vinculin (SC-73614, Santa Cruz Biotechnology Inc., Santa Cruz, CA, USA). Afterwards, blots were incubated for 1 h with horseradish peroxidase-conjugated goat ant-mouse IgG (Biorad, Berkeley, CA, USA) and then revealed by an enhanced chemiluminescence (ECL) system (Thermo Scientific, Rockford, IL, USA) [35]. Densitometry analysis was performed using Image j software.

#### 3.2.7. Statistical Analysis

[^3^H]CP55,940 radioligand competition binding data are provided as % change from maximal ^3^H bound (i.e., 100%). Data for HitHunter cAMP and PathHunter β-arrestin2 data are shown as % of maximal CP55,940 response (i.e., 100%). Estimates of *K*_i_, EC_50_, and *E*_max_ were determined using non-linear regression three-parameters (GraphPad, Prism, v. 9.0). Data are mean with 95% confidence interval (C.I.) (EC_50_) or mean ± SEM, *n* = 3–6 independent experiments performed in duplicate. Statistical analyses were by nonoverlapping C.I. or two-way ANOVA followed by Bonferroni’s *p*ost hoc test. * *p* < 0.05 relative to CP55,940 within assay. The details of statistical analyses performed for SRB assays and densitometry analysis is reported in the figure legends.

## 4. Conclusions

Beyond the well-documented effects of CB2R agonists in inflammatory and neurodegenerative diseases, CB2R has been recently identified as a novel target in xenograft models of neuroblastoma, suggesting its promising role in high-risk neuroblastoma. Additionally, the anti-proliferative activity in neuroblastoma cell lines of the gold standard CB2 agonist JWH-133 has been reported. Here, we explored the anti-cancer activity of novel CB2R agonists addressing their inhibitory effects in neuroblastoma cell lines. We observed that the arrest of cell viability by these novel compounds was mediated by the CB2R. The ERK/MAPK signaling pathway is known to play a role in neuroblastoma, contributing to the progression toward a pro-tumor cell phenotype, and is also involved in resistance to chemotherapy. In SH-SY5Y cells, we observed that **FG158a** was able to modulate ERK1/2 expression and that the effect is mediated by CB2R, thus suggesting that the potential anti-cancer effect of this compound might involve this signaling pathway.

The advantage of the CB2R-selective compounds described here might be the documented anti-inflammatory activity of CB2R agonists. It is well known that inflammation can promote cancer and alteration of the tumor microenvironment contributing to tumor progression, inhibition of apoptosis, induction of angiogenesis, and resistance to chemotherapeutics. The crucial role of CB2R in neurodegeneration and in various types of tumors, due to its involvement in the (neuro)-inflammatory processes, makes highly selective CB2R agonists compelling compounds against neuroblastoma.

## Figures and Tables

**Table 1:** Radioligand binding data, cAMP inhibition, and β-arrestin2 recruitment.

Cmpds	CB2R Ki (nM) ^1^	CB1R Ki (nM) ^1^	cAMP Inhibition		β-Arrestin2 Recruitment	
			EC_50_ (95% C.I., nM)	Emax (% CP55,940) ± S.E.M	EC_50_ (95% C.I., nM)	Emax (% CP55,940) ± S.E.M
CP55,940	34 (2.7–57)	6.6 (2.7–15)	9.4 (3.4–29))	100 ± 6.4	560 (410–760)	100 ± 3.4
FG158a	21 (12–50)	>10,000	>10,000	71 ± 2.5 *	>10,000	46 ± 1.8 *
FG160a	16.5 (10–45)	>10,000	600 (73–870) *	90 ± 3.1	>10,000	41 ± 1.0 *
FG161a	45 (32–75)	>10,000	760 (150–2,900) *	78 ± 11	>10,000	47 ± 1.8 *
LV62	37 (13–89)	>10,000	34 (3.9–220)	110 ± 8.8	63 (49–82) *	65 ± 1.4 *

^1^ For radioligand binding, membranes from CHO cells stably expressing human CB2R or CB1R were treated with 0.1 nM-10 μM compounds in the presence of 0.7 nM [^3^H]CP55,940. Data are expressed as % radioligand bound. Data are fit to a nonlinear regression (three parameter, GraphPad v. 9.0) to determine K_i_ (nM) with 95% confidence interval (C.I.). *n* = 3 independent experiments. For cAMP inhibition and β-arrestin2 recruitment, CHO cells stably expressing human CB2R were treated with 0.1 nM–10 μM compounds for 90 min. Data are expressed as % CP55,940 response and presented in Figure 3A,B. Data are fit to a nonlinear regression (three parameter, GraphPad v. 9.0) to determine EC_50_ (nM) with 95% C.I. and E_max_ (% CP55,940 ± S.E.M.). *n* = 3–6 independent experiments performed in duplicate. * *p* < 0.05 compared to CP55,940 as determined by non-overlapping 95% C.I. or two-way ANOVA followed by Bonferroni’s post hoc analysis.

## Supplementary Materials

The following are available online at https://www.mdpi.com/article/10.3390/molecules27093019/s1, Figures S2–S7: ^1^H and ^13^C spectra of the final compounds.
