# Supplementary material for: Synthesis and In Vitro Characterization of Selective Cannabinoid CB2 Receptor Agonists: Biological Evaluation against Neuroblastoma Cancer Cells
Source: Molecules. 2022 May 7;27(9):3019. doi: 10.3390/molecules27093019 (PMC9101764; doi:10.3390/molecules27093019)

Supporting Information

Synthesis and In vitro Characterization of Selective Cannabinoid CB2 receptor Agonists: Biological Evaluation against Neuroblastoma Cancer Cells

Francesca Gado [1,#,ϕ], Rebecca Ferrisi [1,#], Sarah Di Somma [2], Fabiana Napolitano [2], Kawthar A. Mohamed [3], Lesley A. Stevenson [4], Simona Rapposelli [1], Giuseppe Saccomanni [1], Giuseppe Portella [2], Roger G. Pertwee [4], Robert B. Laprairie [3,5], Anna Maria Malfitano [2,*], Clementina Manera [1,*]

[1] Department of Pharmacy, University of Pisa, Italy; rebecca.ferrisi@phd.unipi.it (R.F.); simona.rapposelli@unipi.it (S.R.); giuseppe.saccomanni@unipi.it (G.S.); clementina.manera@unipi.it (C.M.)
[2] Department of Translational Medical Sciences, University of Naples Federico II, Italy; annamaria.malfitano@unina.it (A.M.M.); giuseppe.portella@unina.it (G.P.); sarah.disomma@unina.it (S.D.S); fabiana.napolitano2@unina.it (F.N.);
[3] College of Pharmacy and Nutrition, University of Saskatchewan, Canada; kam913@mail.usask.ca (K.A.M.); robert.laprairie@usask.ca (R.B.L.);
[4] Institute of Medical Sciences, University of Aberdeen, UK; l.a.stevenson@abdn.ac.uk (L.A.S.); rgp@abdn.ac.uk (R.G.P.);
[5] Department of Pharmacology, College of Medicine, Dalhousie University, Canada; robert.laprairie@usask.ca (R.B.L.);

CONTENTS OF SUPPORTING INFORMATION

S2-S7 ^{1}H- and ^{13}C-NMR Spectra of compounds **FG158a**-*trans*, **FG158a**-*cis*, **FG160a**-*trans*, **FG160a**-*cis*, **FG161a**-*trans*, and **FG161a**-*cis*.

FG158a *cis* ^{1}H-NMR

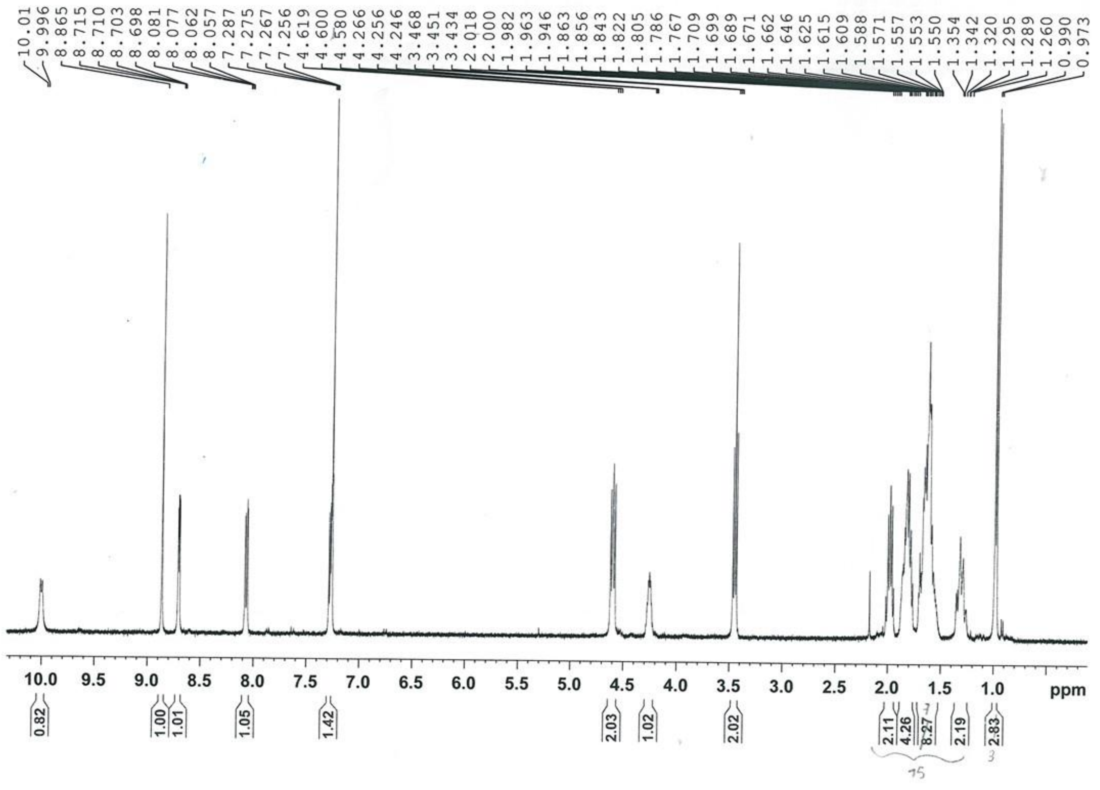

FG158a *trans* ^{1}H-NMR

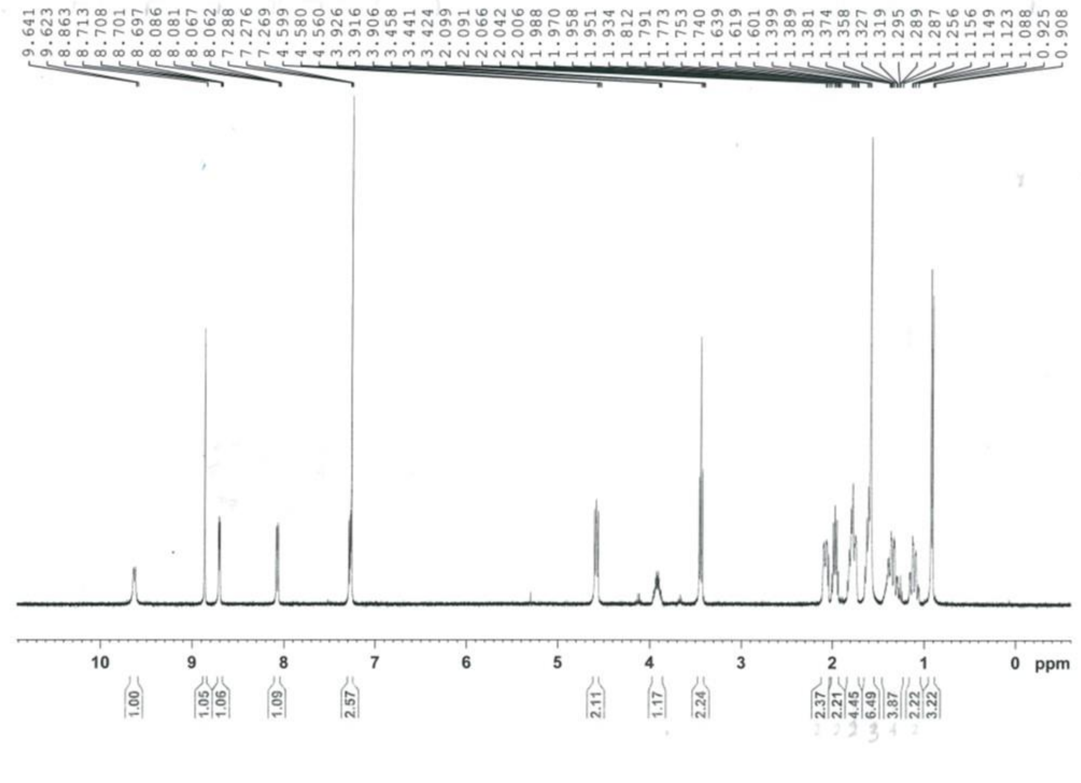

FG158a *cis* ^{13}C-NMR

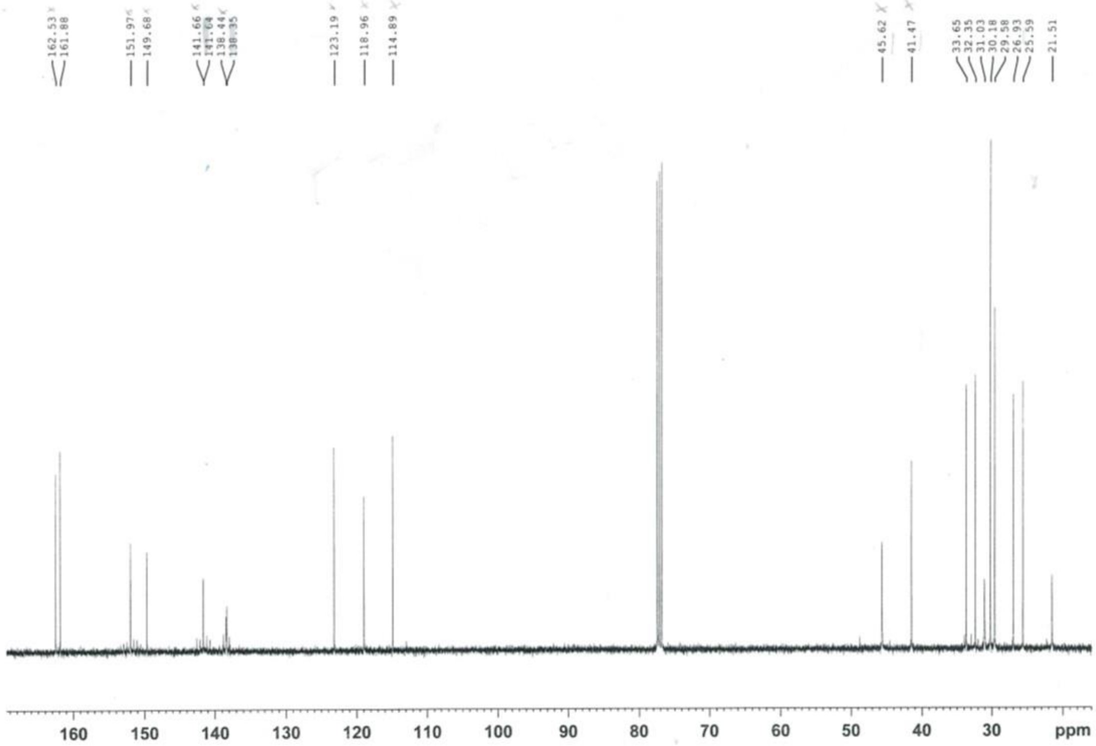

FG158a *trans* ^{13}C-NMR

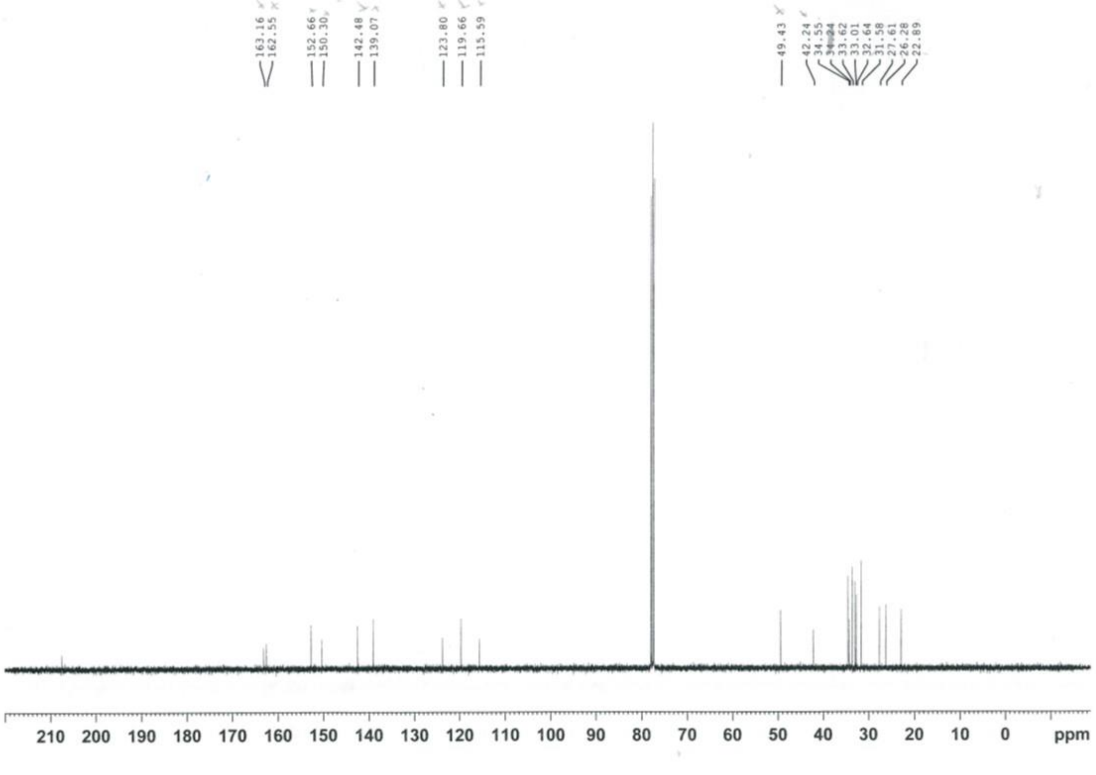

FG160a *cis* ^{1}H-NMR

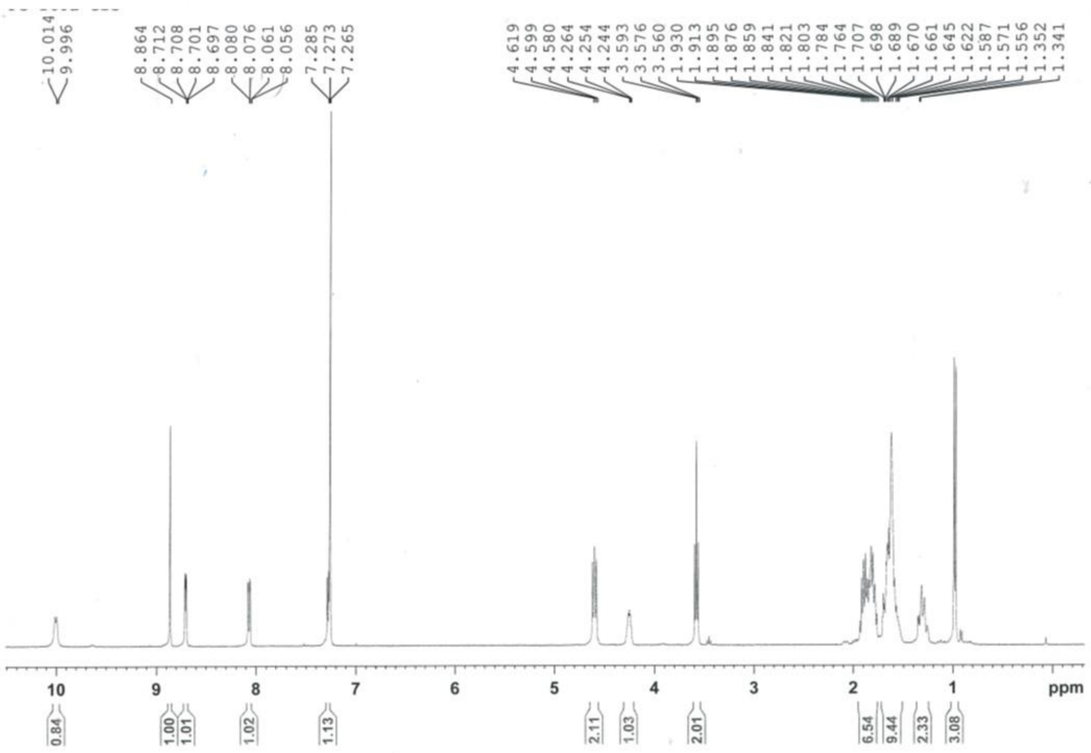

FG160a *trans* ^{1}H-NMR

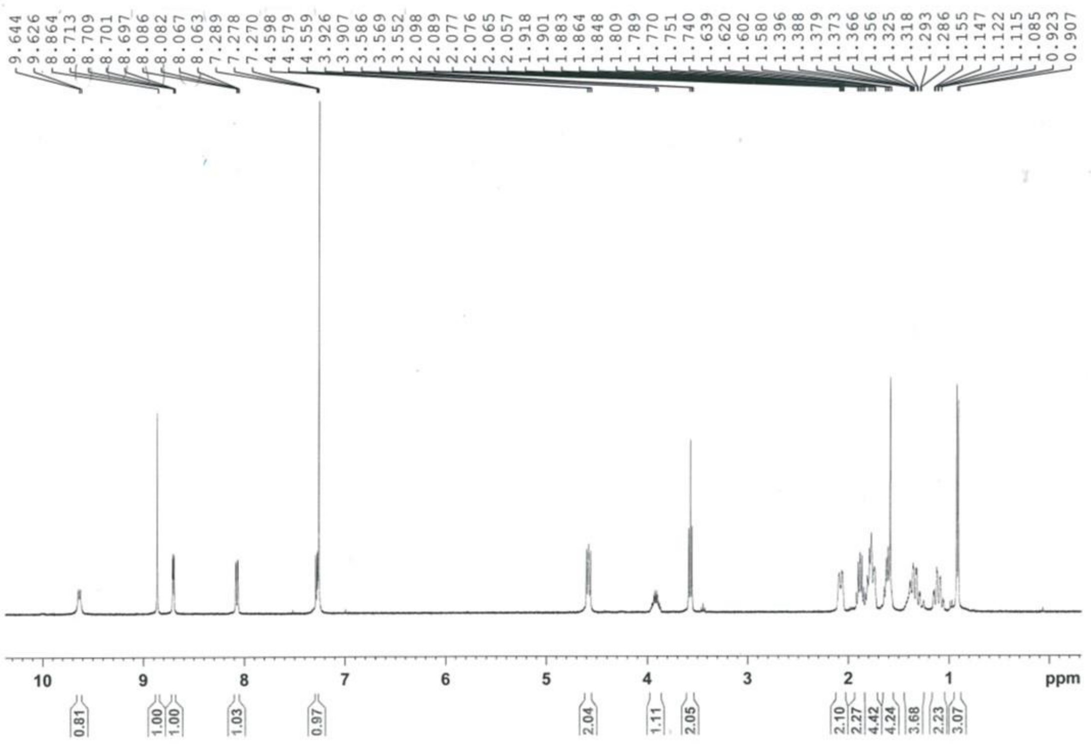

FG160a *cis* ^{13}C-NMR

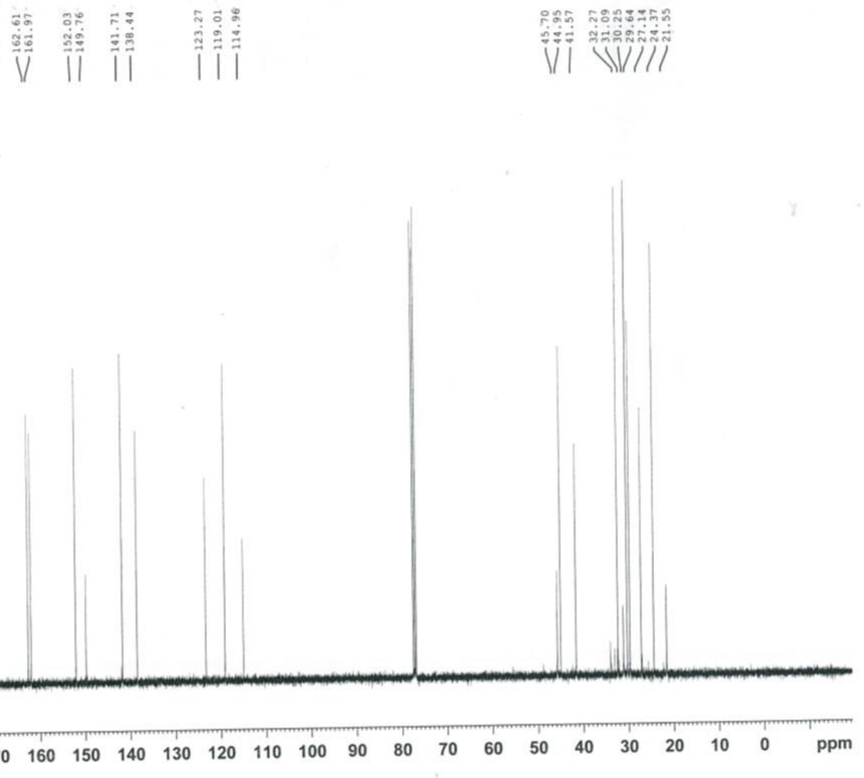

FG160a *trans* ^{13}C-NMR

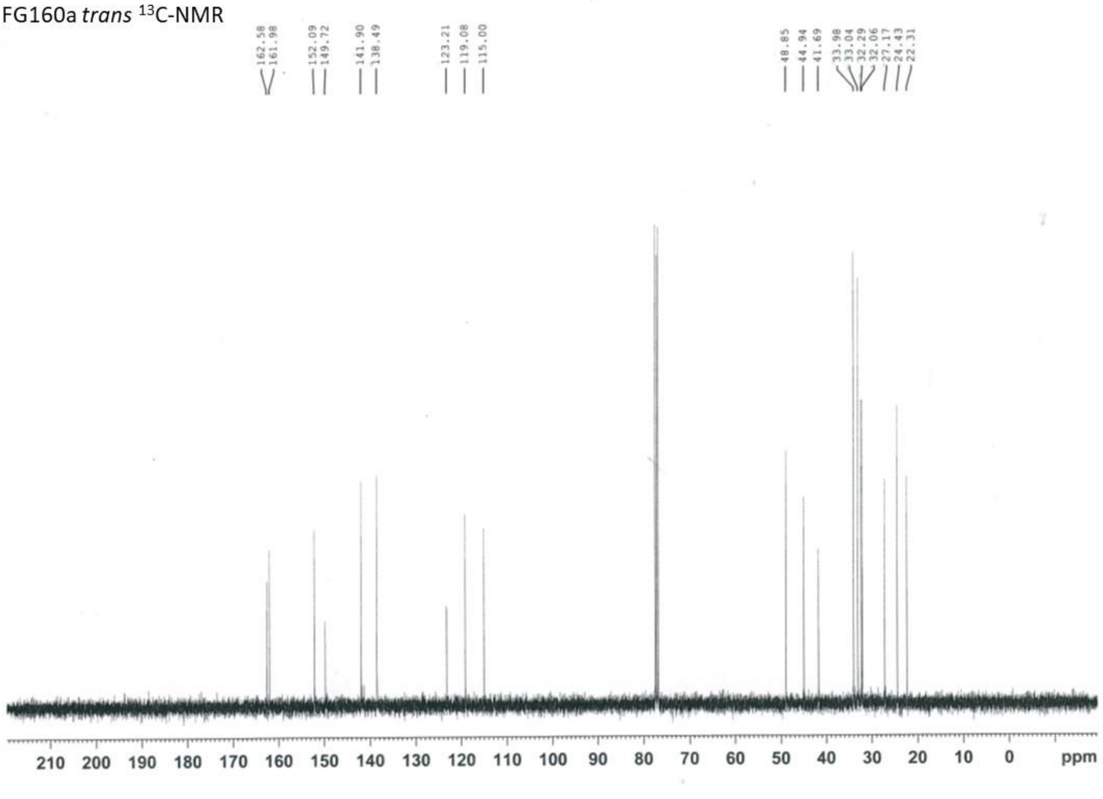

FG161a *cis* ^{1}H-NMR

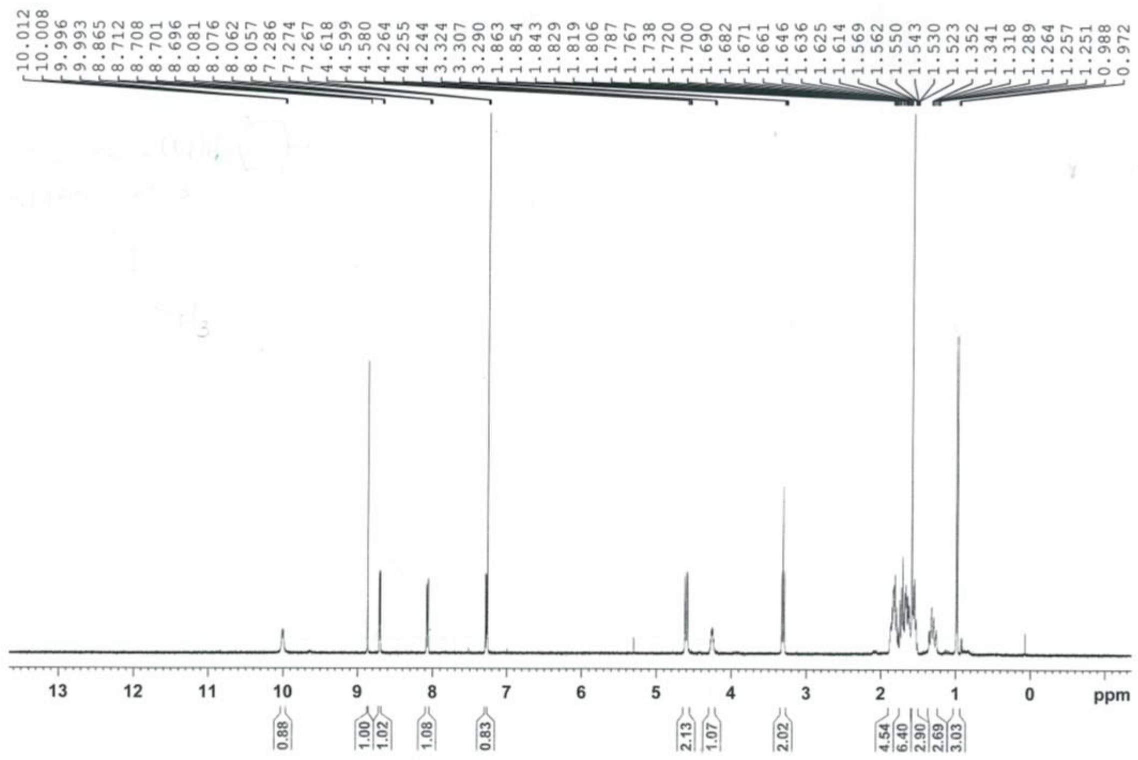

FG161a *trans* ^{1}H-NMR

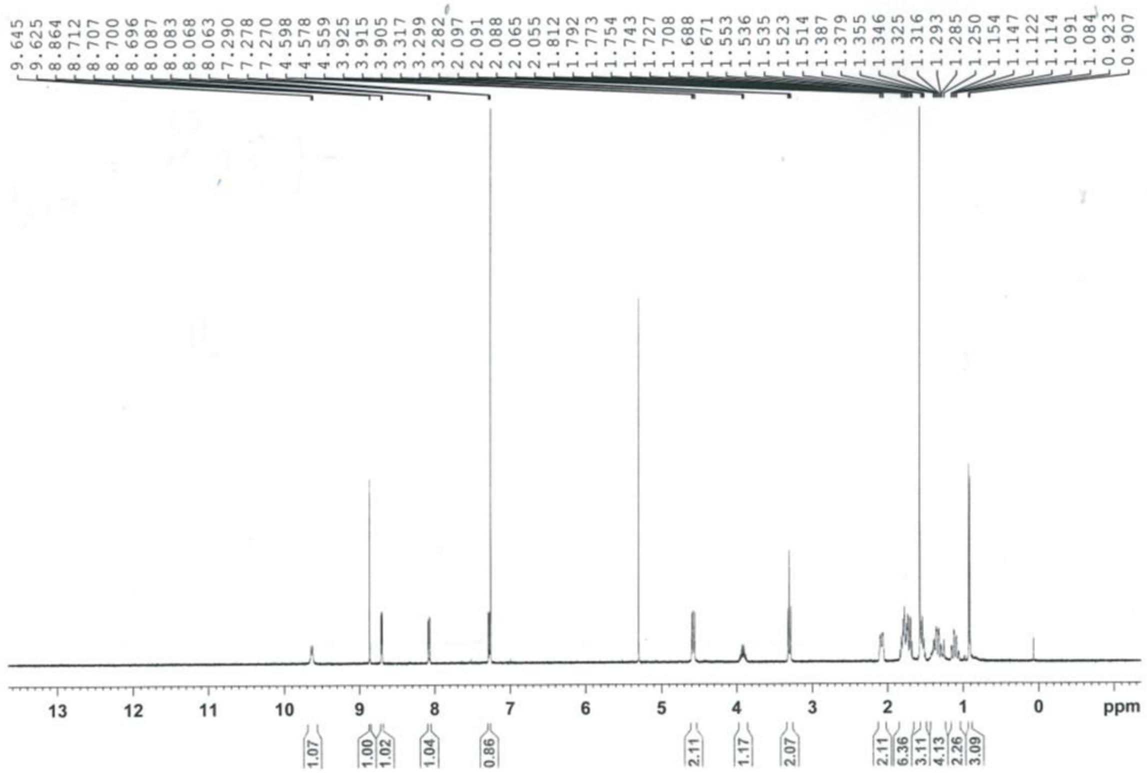

FG161a *cis* ^{13}C-NMR

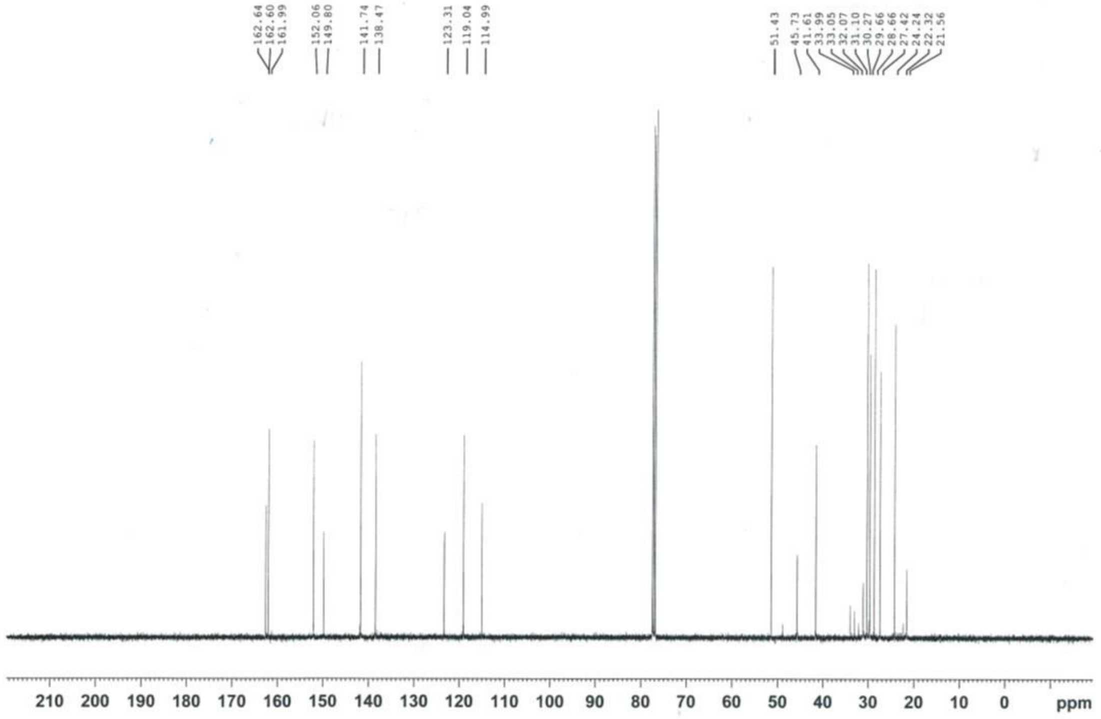

FG161a *trans* ^{13}C-NMR

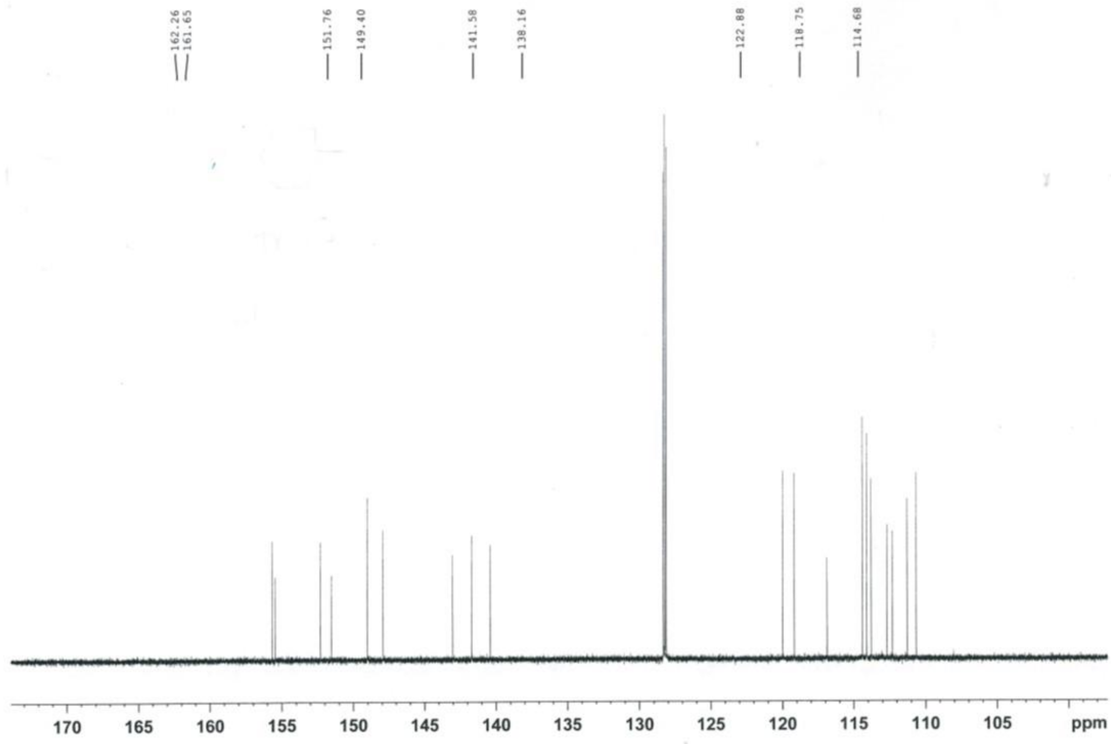

Supplement: Supplementary file 1 [file molecules-27-03019-s001.zip › molecules-1691929-supplementary.pdf]
